# Toceranib phosphate in the management of canine insulinoma: A retrospective multicentre study of 30 cases (2009–2019)

**DOI:** 10.1002/vro2.27

**Published:** 2022-01-20

**Authors:** Sabina Sheppard‐Olivares, Nora M. Bello, Chad M. Johannes, Samuel E. Hocker, Barbara Biller, Brian Husbands, Elizabeth Snyder, Mattison McMillan, Talon McKee, Raelene M. Wouda

**Affiliations:** ^1^ Department of Clinical Sciences College of Veterinary Medicine Kansas State University Manhattan Kansas USA; ^2^ Department of Statistics College of Arts and Sciences Kansas State University Manhattan Kansas USA; ^3^ Department of Clinical Sciences College of Veterinary Medicine Iowa State University Ames Iowa USA; ^4^ Ontario Veterinary College University of Guelph Guelph Ontario Canada; ^5^ Flint Animal Cancer Center College of Veterinary Medicine and Biomedical Sciences Colorado State University Fort Collins Colorado USA; ^6^ Veterinary Clinical Sciences Department College of Veterinary Medicine University of Minnesota St. Paul Minnesota USA; ^7^ Department of Medical Sciences School of Veterinary Medicine University of Wisconsin‐Madison Madison Wisconsin USA; ^8^ Las Vegas Veterinary Specialty Center Las Vegas Nevada USA; ^9^ Clinical Studies Department VCA Inc. Los Angeles California USA; ^10^ Present address: 3901 Guadalupe Street, Austin, TX 78751, USA.; ^11^ Present address: Department of Clinical Sciences, College of Veterinary Medicine, Kansas State University, 1800 Denison Avenue, Manhattan, KS 66506, USA.; ^12^ Present address: 4120 Clydesdale Pkwy, Loveland, CO 80538, USA.; ^13^ Present address: Department of Veterinary Clinical Sciences, College of Veterinary Medicine, The Ohio State University, 1900 Coffey Road, Columbus, OH 43210, USA.; ^14^ Present address: BluePearl Specialty and Emergency Pet Hospital, 1646 Spring Cypress Rd Ste 100, Spring, TX 77388, USA.; ^15^ Present address: College of Veterinary Medicine, Washington State University, Pullman, WA 99164, USA.

## Abstract

**Background:**

Insulinomas are the most common tumour of the endocrine pancreas in dogs. These malignant tumours have a high metastatic rate and limited chemotherapeutic options. The multi‐receptor tyrosine kinase inhibitor sunitinib malate has benefit in the treatment of metastatic insulinoma in people. Toceranib phosphate, an analogous veterinary agent, may provide benefit for dogs.

**Methods:**

A retrospective study describing the extent and duration of clinical outcomes and adverse events (AEs) in dogs diagnosed with insulinoma and receiving toceranib.

**Results:**

Records for 30 dogs diagnosed with insulinoma and having received toceranib were identified from a medical record search of five university and eight referral hospitals. The median progression‐free interval and overall survival time were 561 days (95% confidence interval (CI): [246, 727 days]) and 656 days (95% CI: [310, 1045 days]), respectively. Of the dogs for which the canine Response evaluation criteria for solid tumours tool could be applied, the majority (66.7%) showed either a complete response, partial response or stable disease. Time to clinical progression was associated with prior intervention and type of veterinary practice. Larger dogs were at increased risk for disease progression and death. No novel AEs were reported.

**Conclusions:**

Most dogs diagnosed with insulinoma and receiving toceranib appeared to have a clinical benefit. Randomised, prospective studies are needed to better elucidate and objectively quantify the potential effect and survival benefit of toceranib therapy for management of insulinoma in dogs.

## INTRODUCTION

Insulinomas are insulin‐secreting tumours arising from pancreatic beta cells. Insulinomas are the most common tumour of the endocrine pancreas in dogs.^1^, ^2^, ^3^ They are malignant tumours, with macroscopic metastatic lesions present in approximately 50% of dogs at the time of diagnosis.^1^, ^2^, ^3^, ^4^, ^5^, ^6^, ^7^, ^8^, ^9^ Moreover, it is clinically anticipated that remaining dogs will develop metastasis and/or locoregional recurrence, with associated clinical signs, despite surgery.^1^, ^2^, ^3^, ^6^, ^7^

The most frequent clinical signs in dogs with insulinoma are due to neuroglycopenia, including weakness, ataxia, collapse, disorientation, behaviour changes and seizures.^1^, ^2^, ^3^, ^4^, ^5^, ^6^, ^7^, ^8^, ^9^, ^10^ Immediate treatment aims to correct the hypoglycaemia and ameliorate these clinical signs. Longer term treatment ideally addresses the primary tumour and metastasis.^1^, ^2^, ^3^, ^4^, ^6^

Despite debate around the benefit of surgery for advanced stage disease,^3^, ^10^, ^11^, ^12^ partial pancreatectomy remains the mainstay of treatment.^1^, ^2^, ^3^, ^4^, ^6^, ^8^, ^9^, ^10^, ^11^, ^12^ Survival times between 258 and 785 days are reported for dogs undergoing surgical excision of macroscopic disease.^4^, ^5^, ^6^, ^7^, ^8^, ^9^, ^10^, ^11^, ^12^ Nevertheless, surgery alone is unlikely to be curative as historical outcomes indicate most dogs have at least micrometastasis at diagnosis,^1^, ^2^, ^4^, ^5^, ^8^, ^9^ and disease progression is often life‐limiting.^1^, ^2^, ^3^, ^4^, ^5^, ^6^, ^7^, ^8^, ^9^, ^10^ Moreover, certain cases may not be amenable to surgery due to location and/or extent of disease, inability to localise lesion(s) and/or owner preference.^3^, ^11^, ^12^

Medical management options for dogs with insulinoma include lifestyle and dietary modifications, and/or pharmaceutical management with glucocorticoids, diazoxide, glucagon, octreotide, propranolol, alloxan or streptozocin, with only the latter two being cytotoxic.^1^, ^2^, ^3^, ^4^, ^5^, ^6^, ^8^, ^10^, ^13^, ^14^, ^15^, ^16^, ^17^, ^18^ Drawbacks to these include unpredictable responses, side effects and logistical issues with the route and frequency of administration, product availability and cost.^2^, ^6^, ^15^, ^16^, ^17^, ^18^ There remains a need to identify effective, well‐tolerated and practical medical interventions, to manage clinical signs and disease progression, while maintaining quality of life in dogs with insulinoma.

Sunitinib malate (Sutent®; Pfizer, Inc., New York, NY, USA) is an oral small molecule inhibitor, initially approved by the USA Food and Drug Administration for the treatment of imatinib‐resistant gastrointestinal stromal tumour^19^, ^20^ and advanced renal cell carcinoma in people;^20^, ^21^ additional applications have been identified.^22^, ^23^ Sunitinib was approved for the treatment of locally advanced, or metastatic, pancreatic neuroendocrine tumours (pNETs) in people, a classification encompassing all tumours arising from the multipotent stem cells of the pancreatic ductal epithelium, including insulinoma.^24^, ^25^, ^26^

Toceranib phosphate (Palladia®; Zoetis Animal Heath, Madison, NJ, USA) is a veterinary oral small molecule inhibitor, with similar molecular targets to sunitinib.^27^, ^28^, ^29^, ^30^ While approved for the treatment of mast cell disease,^31^ clinical responses to toceranib have been documented in dogs with a spectrum of solid tumour types, including several of neuroendocrine histology.^29^, ^32^, ^33^, ^34^, ^35^, ^36^, ^37^, ^38^, ^39^, ^40^, ^41^, ^42^ A retrospective study of five dogs diagnosed with metastatic or recurrent insulinoma and treated with toceranib suggested improved outcomes when compared with seven dogs treated palliatively.^43^ A case report describing a dog with metastatic insulinoma reported long‐term glycaemic control with toceranib.^44^

The objectives of this retrospective study were to describe the extent and duration of clinical outcomes and adverse events (AEs), in dogs diagnosed with insulinoma and treated with toceranib.

## MATERIALS AND METHODS

### Case selection

The medical record databases of five collaborating academic and eight referral veterinary hospitals in the US and Canada were searched for cases in which dogs diagnosed with insulinoma were treated with toceranib between June 2009 and March 2019. Inclusion criteria were: (1) documentation of fasting hyperinsulinaemia with paired hypoglycaemia and/or cytological or histological diagnosis of a primary pancreatic tumour with neuroendocrine morphology; (2) reasonable elimination of other causes of hypoglycaemia, as decided by the clinicians primarily responsible for each case and documented in the medical records, typically considering history, physical examination findings, laboratory anomalies or lack thereof and imaging; (3) documentation of any prior, concurrent or subsequent treatments; (4) documentation of toceranib dosage and schedule; (5) at least one documented follow‐up assessment during the toceranib treatment period. Exclusion criteria were: (1) absence of a clinical diagnosis of insulinoma and (2) insufficient details documenting toceranib treatment and follow‐up.

Information collated included: signalment, weight, clinical signs at diagnosis and at subsequent visits, glucose and insulin measurements at diagnosis and subsequently, findings of any imaging performed at diagnosis and subsequently, histological and/or cytological diagnoses, details of any clinicopathologic analyses performed at baseline or subsequently, blood pressure measurement at diagnosis, toceranib dose and administration regimen, response to treatment, AEs, duration of toceranib therapy, reason for cessation, any anticancer therapy after toceranib, comorbidities, concomitant medications, and the date and reason for death or euthanasia. Additional details were obtained from the referring veterinarian(s) and/or owner(s) where necessary.

End points were progression‐free interval (PFI) and overall survival time (OST). The PFI was defined as the interval in days from the date of toceranib initiation to the date of documented clinical progression (CP). The CP was defined as either the return of clinical signs associated with neuroglycopenia as reported by the overseeing clinician(s) and/or owner(s), loss of glycaemic control based upon serial fasting blood glucose measurements, or the development of novel metastasis, metastatic progression and/or local progressive disease (PD) according to the canine Response evaluation criteria for solid tumours (cRECIST),^45^ applied based upon repeated abdominal ultrasound, CT and/or thoracic radiographs when available. For cases in which CP was not documented, PFI was considered right‐censored and defined as interval in days to date of last data submission or date of death or euthanasia. The OST was defined as the interval in days from the date of diagnosis to the date of death or euthanasia. For cases for which death or euthanasia was not recorded, OST was considered right‐censored and defined as the interval from the date of diagnosis to the date of last data submission.

As in previous studies, stage was retrospectively assigned according to the World Health Organization (WHO) Tumour Node Metastasis (TNM) system.^4^, ^7^, ^10^ With stage I being disease confined to the pancreas, stage II indicating lymph node metastasis and stage III indicating distant metastasis.^46^ The WHO recommendations advise lymph node examination by laparotomy or laparoscopy, however, the present study recognised non‐invasive assessments, such as ultrasound and CT, as applicable.^10^

The AEs were retrospectively graded according to the Veterinary Co‐operative Oncology Group – Common Terminology Criteria for Adverse Events (VCOG‐CTCAE) following chemotherapy or biological antineoplastic therapy in dogs and cats.^47^

### Statistical analyses

The Kaplan–Meier estimator was applied to estimate the survival distribution of the two continuous right‐censored response variables, PFI and OST. Censoring criteria for PFI and OST were as previously defined. Computations were conducted using the LIFETEST procedure of SAS (Version 9.4, Cary, NC, USA). Confidence intervals (CIs) at given time points were calculated based on log–log transformations. A Cox proportional hazards (PH) model was fitted to each response variable, PFI and OST. The linear predictor in the PH model evaluated several, non‐time‐dependent, explanatory covariates as potential risk factors, specifically sex, age at diagnosis, weight at diagnosis, type of veterinary practice, tumour stage, therapy prior to toceranib, toceranib dose, dosing regimen and all two‐way interactions. Selection of covariates into the model was conducted by stepwise selection at a 10% significance level for entry and 15% for removal. For each model, the PH assumption was evaluated. Computations were conducted using the PHREG procedure of SAS (Version 9.4).

## RESULTS

### Case population

The study population included 30 dogs; eight mixed breed dogs, two Boston terriers, two Chihuahuas, two Labrador retrievers and one of each of the following breeds: Afghan hound, Australian shepherd, chow chow, cocker spaniel, coonhound, dachshund, Doberman pinscher, Irish setter, Jack Russell terrier, papillon, Pekingese, Pomeranian, Scottish terrier, shar pei, West Highland white terrier and Yorkshire terrier. There were 14 neutered male (46.7%) and 16 spayed female (53.3%) dogs. The median age at diagnosis was 9 years (min—max: 5–15 years). The median weight was 14.9 kg (min—max: 2.9–44.4 kg).

### Presentation, diagnosis and staging

The majority of dogs (*n* = 25/30, 83%) initially presented with clinical signs attributable to neuroglycopenia, including seizures (*n* = 13), collapse (*n* = 7), ataxia (*n* = 5), muscle tremors (*n* = 3) and twitching (*n* = 3). Two dogs presented with lethargy and two with vomiting. Four dogs had hypoglycaemia incidentally identified on blood tests, although were without clinical signs. Three of these dogs were presented for routine annual examination and the fourth for a subcutaneous mass.

A histopathological or cytological diagnosis was available for 25/30 dogs. Twenty‐one dogs underwent partial pancreatectomy with histopathology. In four dogs, ultrasound‐guided fine needle aspirate biopsy (FNA) of a pancreatic mass was performed, with cytology. All 25 dogs were also hypoglycaemic; 23/25 dogs had an inappropriate fasting insulin level in the presence of hypoglycaemia. Of the remaining 5/30 dogs, four were diagnosed based on an inappropriate fasting insulin level in the presence of hypoglycaemia, combined with an imaging findings of a pancreatic nodule on CT (*n* = 2) or ultrasound (*n* = 2), with no pathological confirmation. One dog was diagnosed based on clinical signs consistent with neuroglycopenia, combined with an inappropriate fasting insulin level in the presence of hypoglycaemia. In all cases, alternative causes of hypoglycaemia, including idiopathic in toy breed dogs, sepsis, hepatic dysfunction, renal failure, adrenocortical insufficiency, toxin exposure and extrapancreatic tumour‐associated paraneoplastic hypoglycaemia, were considered and reasonably eliminated.

All dogs were hypoglycaemic at the time of diagnosis. Although the normal reference range varied for each laboratory evaluating the fasting blood glucose concentrations, all laboratories asserted values below 3.33 mmol/L were abnormal. Twenty‐eight dogs had fasting hypoglycaemia in the presence of a normal or increased insulin level. Although the normal insulin reference range also varied for each laboratory evaluating it, all laboratories asserted values between 14 and 140 pmol/L to be normal and above 140 pmol/L abnormal. Insulin levels were not measured in the remaining two dogs, which were diagnosed based upon histopathology and/or consistent cytology. The median fasting blood glucose concentration at diagnosis was 2.16 mmol/L (1.89–3.27 mmol/L) and the median fasting insulin concentration was 101.8 μIU/ml (14.6–647.3 μIU/ml).

Based on the diagnostic tests performed, eight dogs had WHO TNM stage I, 13 stage II and nine stage III disease.^45^ Imaging tests performed included abdominal ultrasound (*n* = 22), thoracic radiographs (*n* = 21) and/or abdominal ± thoracic CT (*n* = 12). Imaging findings and their clinical sequelae are reported in Table 1. Metastatic locations confirmed by FNA and cytology, or tissue biopsy and histopathology, included the locoregional pancreatic lymph nodes (*n* = 13), liver (*n* = 6), distant lymph nodes (*n* = 2), hepatic lymph nodes (*n* = 3) and spleen (*n* = 1). Some of these dogs had imaging abnormalities as documented in Table 1. For eight of the dogs with lymph node metastasis confirmed by histopathology and two dogs with histopathologically confirmed liver metastasis, prior imaging was not suggestive of metastasis. Moreover, not all imaging anomalies were further investigated (Table 1).

**TABLE 1:** Summary of imaging tests undertaken in dogs diagnosed with insulinoma and commencing toceranib therapy, imaging findings, any further investigations then undertaken and results consistent with neuroendocrine disease

Staging test and reported findings	Number of cases undergoing staging tests and abnormalities reported (*n*/30)	Number of these cases undergoing further investigation of potential disease and/or metastasis	Number of these cases with cytologically and/or histologically confirmed neuroendocrine disease
Thoracic radiographs	21	NA	NA
No significant findings	21	NA	NA
Abdominal ultrasound	22	18	14
At least one hypoechoic pancreatic nodule	12	7	7
Locoregional pancreatic lymphadenopathy	4	3	3
Non‐regional lymphadenopathy	1^a^	1^a^	0
Multiple hypoechoic hepatic nodules	5	4^b^	3^b^
Multiple hypoechoic splenic nodules	4	2^c^	0^c^
Adrenal mass	1	0	0
Cranial abdominal mass	1	1	1
Thickened intestinal walls	2	0	0
No significant findings	5	3^d^	3^d^
Computed tomography (abdomen ± thorax)	12	8	8
Pancreatic nodules with arterial phase enhancement	11	8	8
Locoregional pancreatic lymphadenopathy	5	5	5
Hypoattenuating hepatic nodules with venous contrast enhancement	6	5	5
At least one hypoattenuating splenic nodule	2	2	1
Subcutaneous mass	1^e^	1	0
Lung nodule	1	0	0
No significant findings	1	NA	NA

^a^ Determined to be mast cell tumour metastasis via fine needle aspirate (FNA) and cytology.

^b^ One of the four cases was determined to be metastatic mast cell disease via FNA and cytology.

^c^ Determined to be metastatic mast cell disease in one case and lymphoid proliferation in the other via FNA and cytology.

^d^ Three of five cases underwent surgical tissue biopsies confirming the primary pancreatic disease and locoregional lymph node metastasis.

^e^ Diagnosed as a narrowly excised high‐grade soft tissue sarcoma via subsequent histopathology.

See Supporting Information for details on haematological and biochemical analyses, co‐morbidities and concurrent treatments.

### Treatment

Twenty‐one dogs underwent partial pancreatectomy prior to toceranib. One of these dogs underwent a second surgery for PD, 2 years after the initial surgery, then received toceranib. One dog underwent regional lymph node extirpation at the time of partial pancreatectomy. Reasons for toceranib therapy for these 21 dogs that included regional lymph node metastasis present at diagnosis (*n* = 8), hepatic ± lymph node metastasis at diagnosis (*n* = 7), inability to successfully excise the pancreatic mass (*n* = 2), recurrence of clinical signs associated with hypoglycaemia (*n* = 3) and a recurrent pancreatic nodule 1458 days following partial pancreatectomy (*n* = 1). The median duration of time between the initial surgery and starting toceranib was 93 days (min–max: 4–1458 days).

Three of the aforementioned dogs received adjuvant cytotoxic chemotherapy. One of these dogs commenced toceranib 21 days after completing an adjuvant course of four doses of doxorubicin (30 mg/m^2^ intravenously (IV) every 3 weeks), one received two adjuvant doses of streptozotocin (500 mg/m^2^ IV diluted in 225 ml NaCl (0.9%) every 3 weeks) with prednisone before treatment was changed to toceranib and another dog received six adjuvant doses of vinorelbine (15 mg/m^2^ IV every 10–14 days) before commencing toceranib.

Nine dogs did not undergo partial pancreatectomy. Six of these dogs received prednisone prior to toceranib. One of these dogs briefly received glucagon with prednisone, although clinical signs persisted and the glucagon was ceased after only 4 days. All dogs receiving prednisone were started with toceranib when persistence, or recurrence, of clinical signs associated with hypoglycaemia were observed. The median duration between diagnosis and toceranib treatment for these six dogs was 50 days (min–max: 13–453 days). Three of these nine dogs did not receive any treatment prior to commencing toceranib. The median duration between diagnosis and starting toceranib treatment in these dogs was 13 days (min–max: 0–22 days).

The median starting dose of toceranib was 2.67 mg/kg (min–max: 2.1–3.27 mg/kg) by mouth (PO). Twenty‐four dogs initially received toceranib on a Monday, Wednesday, Friday (MWF) schedule and six dogs according to every other day (EOD) schedule. The overall median starting dose intensity was 8.1 mg/kg/week (min–max: 6.30–11.38 mg/kg/week). For the dogs initially receiving toceranib on a MWF schedule (*n* = 24), the median starting dose intensity was 7.92 mg/kg/week (6.30–9.81 mg/kg/week). For the dogs initially receiving toceranib according to an EOD schedule (*n* = 6), the median starting dose intensity was 10.10 mg/kg/week (min–max: 8.40—11.38 mg/kg/week).

The AEs likely or potentially associated with toceranib therapy are detailed in Table 2. Fourteen dogs (46.7%) were reported to develop at least one, grade 1 or 2, self‐limiting haematological AE during toceranib therapy. Biochemical anomalies were documented in 17 dogs (56.7%). Seventeen dogs (56.7%) were reported with one or more gastrointestinal AEs. The majority of these were grade 1 or 2, although a dose reduction was instituted for 14 dogs (46.7%) due to gastrointestinal AEs.

**TABLE 2:** Dogs diagnosed with insulinoma and records of adverse events likely or potentially attributable to toceranib phosphate therapy

Adverse event	Grade 1 *n* (%)	Grade 2 *n* (%)	Grade 3 *n* (%)	Grade 4 *n* (%)	Grade 5 *n* (%)
**Gastrointestinal**					
Anorexia	9 (30)	1 (3.3)	1 (3.3)	–	–
Diarrhoea	7 (23)	–	–	–	–
Haematochezia	3 (10)	–	–	–	–
Vomiting	5 (17)	1 (3.3)	–	–	–
Gastric ulceration	–	–	–	–	1^a^ (3.3)
**Constitutional**					
Fever	1 (3)	–	–	–	–
Weight Loss	2 (6)	–	–	–	–
Lethargy	2 (6.7)	1 (3.3)	–	–	–
**Haematological**					
Anaemia	5 (16.6)	–	–	–	–
Neutropenia	7 (23.3)	2 (6.7)	–	–	–
Thrombocytopenia	1 (3.3)	–	–	–	–
Thrombocytosis	2 (6.7)	–	–	–	–
**Biochemical**					
Azotaemia	2 (6.7)	–	–	–	–
Increased blood urea nitrogen	2 (6.7)	–	–	–	–
Increased alkaline phosphatase	7 (23.3)	2 (6.7)	3 (10.0)	–	–
Increased alanine aminotransferase	5 (16.7)	4 (13.3)	2 (6.7)	–	–
Increased aspartate aminotransferase	2 (6.7)	2 (6.7)	–	–	–
Hypoalbuminaemia	1 (3.3)	–	–	–	–
Increased cholesterol	1 (3.3)	–	–	–	–
Hypocalcaemia	1 (3.3)	–	–	–	–
**Cardiovascular**					
Hypertension	2 (6.7)	1 (3.3)	–	–	–
**Renal**					
Proteinuria	2 (6.7)	1 (3.3)	2 (6.7)	–	–

*Note*: Cases were graded according to the Veterinary Co‐operative Oncology Group – Common Terminology Criteria for Adverse Events (VCOG‐CTCAE) following chemotherapy or biological antineoplastic therapy in dogs and cats v1.1.^31^

^a^ Suspected clinically, but not confirmed with postmortem examination.

Twenty‐three dogs were concurrently administered prednisone at a median dose of 0.5 mg/kg PO once daily (0.5–0.8 mg/kg). Eleven of these dogs were receiving prednisone, with inadequately controlled disease, at the time of commencing toceranib. Twelve dogs were commenced on prednisone around the time of commencing toceranib. Two of these dogs received both prednisone and diazoxide (5–10 mg/kg PO twice daily) during treatment with toceranib.

### Clinical response

All dogs were treated in the setting of documented PD or with CP as defined in the section Materials and Methods. This included three dogs that had undergone successful partial pancreatectomy, with no appreciable residual disease, who then developed recurrent clinical signs attributed to hypoglycaemia.

Response criteria as defined by cRECIST were able to be assigned for 15 dogs;^45^ based upon repeated imaging, abdominal ultrasound (*n* = 13) ± thoracic radiographs (*n* = 6) or CT‐scan (*n* = 2). Intervals between assessments were not standardised and intervals were documented between 1 week and 2 months. Of the dogs with a cRECIST response reported, 66.7% of dogs showed a clinical benefit; six (40%) dogs showed a complete response (CR), one (6.7%) dog had a partial response (PR), three (20%) dogs had stable disease (SD) and five (33.3%) dogs had PD. All dogs that had a CR, PR or SD were also reported to be normoglycaemic at each response assessment.

Twelve dogs were monitored based on clinical signs and repeated blood glucose measurements. Intervals between these assessments were not standardised and even the monitoring intervals for each individual dog varied as treatment time progressed, however, the intervals were documented to be between 1 week and 2 months. The median duration of reported normoglycaemia and lack of associated clinical signs was 275 days (min–max: 12–727 days).

Three dogs did not have repeated imaging or blood glucose measurements; they were re‐evaluated based solely on clinical signs associated with hypoglycaemia. One of these dogs was reported to have CP after just 25 days of toceranib treatment, one dog was reported to have CP after 74 days and one dog was reported to have CP after 258 days. In these cases, the intervals were dependent upon the owners’ reporting.

The median duration of toceranib treatment for all dogs was 281 days (min–max: 10–727). For 22 dogs toceranib was stopped. Nineteen dogs were discontinued because of CP; including one dog after 883 days due to an unspecified increase in liver enzyme values, another dog because of financial concerns, another dog after 658 days, because of a persistent absence of CP, but then was recommenced on toceranib when CP was documented another 338 days later. One dog had CP, specifically splenic metastasis confirmed by FNA after 288 days, yet continued to receive toceranib. Eight dogs remained on toceranib without documentation of CP until study conclusion (*n* = 7) or death (*n* = 1) – this dog died acutely 154 days after commencing toceranib, presumably as a consequence of gastrointestinal ulceration, although no postmortem examination was performed.

One dog that developed CP subsequently received masitinib (∼13.5 mg/kg PO once daily). However, the dog's clinical signs acutely worsened, masitinib was stopped and metronomic cyclophosphamide (15 m/m^2^ once daily) administered for 6 days before the dog was euthanased. One dog that developed CP then received two doses of streptozotocin (500 mg/m^2^ IV every 3 weeks) before therapy was ceased. Four dogs were continued on prednisone until euthanasia (*n* = 3) or being lost to follow up (*n* = 1).

### Time‐to‐event outcomes

**FIGURE 1:**
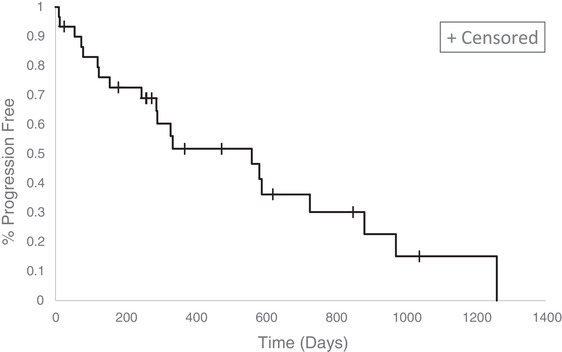
Estimated Kaplan–Meier survival curve for median overall progression‐free interval for 30 dogs with insulinoma treated with toceranib. In absence of documentation of progressive disease, disease progression was considered not observed for 10 dogs, up until the last veterinary visit and the progression‐free interval was right‐censored for these cases

The CP was documented in 20 dogs (66.6%). For the 10 dogs without documented CP, PFI was right‐censored at the time of ceasing toceranib because of AEs (*n* = 3) or the last veterinary visit (*n* = 7). The median PFI was 561 days (95% CI: [246, 727 days]) (Figure 1). The probability of a dog being free of CP by 100, 250 and 500 days was estimated at 0.83 ± 0.07 (±standard error), 0.69 ± 0.09 and 0.52 ± 0.10, respectively.

**FIGURE 2:**
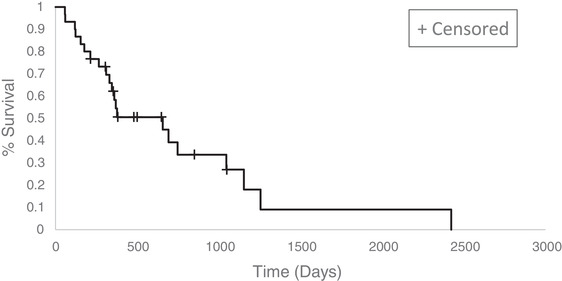
Estimated Kaplan–Meier survival curve for median overall survival time for 30 dogs with insulinoma treated with toceranib. In the absence of a death record, the overall survival time was right‐censored for nine dogs that were alive and still receiving toceranib at the time of last assessment

Death attributed to CP was reported for 20 dogs, although details were lacking for five. One dog died of complications of presumptive gastrointestinal ulceration, 154 days after toceranib initiation, although not confirmed by postmortem examination. In the absence of a death record, OST was right‐censored for nine dogs that were alive and receiving toceranib at the time of last contact. The median OST was 656 days (95% CI: [310, 1045 days]) (Figure 2). The probability of survival to 100, 250, 500 and 1000 days was estimated at 0.93 ± 0.05, 0.77 ± 0.08, 0.51 ± 0.10 and 0.34 ± 0.10, respectively.

Of the explanatory covariates examined with respect to PFI, the stepwise model selection approach indicated statistical evidence for a significant association between PFI and several covariates considered jointly; therapy prior to toceranib (*p* = 0.0050), type of practice (*p* = 0.0025) and weight at diagnosis (*p* = 0.0301). After accounting for type of veterinary practice and weight, dogs that received prior therapy showed an estimated hazard ratio (HR) for PD of 8.4 (95% CI: [2.4, 41.4]), relative to dogs that did not. After adjusting for prior therapy and weight, dogs treated at an academic institution showed an estimated HR for CP of 4.6 (95% CI: [1.5, 14.2]) relative to dogs treated in referral veterinary practice. After accounting for the aforementioned covariates, every 1 kg increase in body weight increased the hazard of CP by an estimated multiplier of 1.045 (95% CI: [1.003, 1.084]). There was no statistical evidence of any association between PFI and any of the other proposed covariates at a 5% level of significance (*p* > 0.17).

Of the covariates examined with respect to OST the only explanatory variable identified was weight at diagnosis. For every 1 kg increase in body weight, there was an increased hazard of death by an estimated multiplier of 1.050 (95% CI: [1.012, 1.090]). There was no statistical evidence of any association between OST and any of the other proposed covariates at a 5% of significance (*p* > 0.18).

## DISCUSSION

In this study, improvement in clinical signs attributed to hypoglycaemia was observed in all dogs diagnosed with insulinoma and treated with toceranib. For all 30 dogs, the median PFI and OST were 561 days (95% CI: [246, 727 days]) and 656 days (95% CI: [310, 1045 days]), respectively. Ten of 15 dogs (66.7%), for which cRECIST could be applied, had reported either a CR, PR or SD. Toceranib was generally well‐tolerated, with a comparable AE profile to previous reports and no novel AEs.^27^, ^29^, ^31^, ^32^, ^33^, ^34^, ^35^, ^36^, ^37^, ^38^, ^39^, ^40^, ^41^, ^42^

Prior studies evaluating risk factors for measures of outcome in dogs with insulinoma have found stage to be prognostic,^4^, ^12^, ^48^, ^49^ however, stage was not significantly associated with PFI or OST in this study. As with other studies in which stage was not significant,^10^ the inconsistency is likely a consequence of the retrospective nature of the study, in combination with a limited size of the study population, thereby precluding powerful statistical comparisons between sub‐groups. Additionally, the retrospective application of the WHO TNM stage, based upon the available case information, may have underestimated some stages.

In this study, dogs that received therapy for insulinoma prior to toceranib had a greater hazard for PD than dogs receiving toceranib as first‐line treatment. It is possible that this finding is just a consequence of the retrospective nature of the study data, specifically reflecting an inherent case selection bias and should not be attributed any cause–effect meaning. A formal assessment of cause and effect would require a follow up experimental study in which dogs are randomly assigned to treatment schemes to avoid confounding effects. In this retrospective study, the dogs that had received prior interventions, with PD being the reason for then commencing toceranib, may have had intrinsically resistant disease or disease now selected for resistance mechanisms. Other potential confounding effects, known or unknown, cannot be refuted either based on the observational nature of the data. This finding should be further considered alongside other studies that have demonstrated medical management alone to be a poor prognostic factor,^6^, ^8^ as well as those that have described its potential benefit.^10^

In this study, dogs treated at university hospitals had an increased hazard for CP, relative to those treated at referral hospitals. The clinical relevance of this observation is speculative and the finding likely impacted by biases related to the retrospective nature of the study. Possible explanations include academic institutions pursuing more comprehensive follow‐up and/or documentation, or cases with more advanced disease stages being more commonly referred to academic institutions. Outcomes for other cancers have been associated with practice type. However, previous studies describing treatment and outcomes for canine insulinoma have been largely undertaken through academic sites and it would be irresponsible to draw conclusions based on this retrospective study alone.^4^, ^5^, ^7^, ^8^, ^9^, ^12^ Prospective, randomised or controlled cohort studies, would be required to legitimately compare different treatment settings and to better evaluate the clinical implications for dogs with insulinoma.

There was an increased hazard of both CP and death for every 1 kg increase in body weight at diagnosis. Insulinomas are commonly reported in medium and large breed dogs, yet body weight has not been previously described as prognostic factor.^1^, ^3^, ^4^, ^6^, ^8^, ^12^ A potential impact of body size and obesity on treatment and outcomes warrants further investigation.

Similar to previous reports,^29^, ^31^, ^32^, ^33^, ^34^, ^35^, ^36^, ^37^, ^38^, ^39^, ^40^, ^41^, ^42^ gastrointestinal AEs were the most common AE reported in this study. The majority of gastrointestinal AEs were grade 1 or 2, although dosing modifications were necessary in 14 dogs. Moreover, although dose interval was not associated with outcome, the dog receiving the highest dose and dose intensity of toceranib (3.27 mg/kg PO EOD), while showing a CR, did present on an emergency basis with suspected gastrointestinal perforation. Although postmortem examination was not performed this was reasonably assumed to be a treatment‐associated grade 5 AE. This case highlights that the labelled dose of toceranib causes more marked AEs in dogs,^29^, ^30^ and also emphasises the importance of the studies demonstrating equivalent biological activity and comparable clinical responses with doses of toceranib lower than the labelled dose.^29^, ^50^

Additional AEs included grades 1–3, constitutional, haematological and biochemical AEs (Table 2), which were not associated with overt clinical signs, nor necessitated dosing modifications. At least some of the biochemical AEs were likely the result of pre‐existing conditions based upon comparison with biochemical values obtained prior to commencing toceranib. Ultimately, the AE profile of toceranib, at an appropriate dose, seems more tolerable than that described for alternative cytotoxic agents, considered for the management of canine insulinoma.^14^, ^16^

In people, there are reports of diabetic and non‐diabetic patients experiencing alterations in blood glucose levels, including hyperglycaemia and hypoglycaemia, with various receptor tyrosine kinase inhibitors (RTKIs), including sunitinib.^51^, ^52^, ^53^ The mechanism by which RTKIs affect glucose homeostasis remains unclear.^51^, ^52^, ^53^ While the authors are unaware of documented hypoglycaemia associated with toceranib administration in dogs, it cannot be excluded that dogs may benefit because of an impact on glucose homeostasis. Furthermore, it is important for clinicians to be aware of the hypothetical risk of iatrogenic hypoglycaemia or hyperglycaemia, when electing toceranib for the management of insulinoma, because it has the potential to obscure treatment response, especially if response assessment is based solely on glycaemic control and clinical signs.

This study has several limitations, largely due to its retrospective and multicentre design. The diagnostic, staging and monitoring tests, and resultant clinical decisions, were not standardised and were doubtlessly influenced by the overseeing clinicians’ and owners’ preferences and even finances. While arguably not ideal, this approach recapitulates clinical practice and is consistent with previous insulinoma studies.^1^, ^2^, ^39^ Re‐staging tests and intervals were also not uniformly performed. While response assessments and measures of outcome would ideally be based upon the routine re‐evaluation of clinical signs associated with hypoglycaemia, repeated blood glucose measurements and imaging enabling the designation of cRECIST, this was not always the case. Most importantly, the PFI and OST may have been extended by the administration of supportive medications. Owner decisions regarding pursuing ongoing treatment and euthanasia are influenced by a spectrum of subjective factors, including perceived quality of life, which in turn may be impacted by supportive medications. Finally, several dogs received concurrent prednisone. While a statistical association was not detected, previous studies have described benefit with prednisone administration;^1^, ^2^, ^3^, ^5^, ^8^ an impact cannot be discounted.

## CONFLICTS OF INTEREST

The authors declare they have no conflicts of interest.

## Supporting information

Supporting Information
S1 Further information results – Presentation, diagnosis and staging
Haematological analyses were available for 25 dogs, and serum chemistry panels for 29, at the time of toceranib initiation. The haematological changes reported were all grade 1, including anaemia (*n* = 3), haemoconcentration (*n* = 1), neutropenia (*n* = 1), neutrophilia (*n* = 5), lymphopenia (*n* = 5), monocytosis (*n* = 1), thrombocytopenia (*n* = 1) and thrombocytosis (*n* = 2). The most commonly reported biochemical abnormality was hypoglycaemia (*n* = 25). Other biochemical changes reported included; variable elevations in alanine transferase (*n* = 4, grade 1 *n* = 2, grade 2 *n* = 2), variable elevations in alkaline phosphatase (*n* = 9, grade 1 *n* = 1, grade 2 *n* = 2, grade 3 *n* = 1, grade 4 *n* = 1 and ungraded *n* = 4), grade 1 hypercholesterolaemia (*n* = 2), grade 2 elevated blood urea nitrogen (*n* = 1), grade 1 elevated creatinine (*n* = 1), ungraded hypophosphataemia (*n* = 2), grade 1 hypokalaemia (*n* = 2) and ungraded elevations in both amylase (n = 4) and lipase (n = 2). Concurrent urinalyses were available for 14 dogs. Two dogs were hyposthenuric and one dog isosthenuric. No dogs were proteinuric. Six dogs had systolic blood pressure measurement performed. Three were hypertensive (systolic blood pressure >140 mmHg).
Comorbidities included chronic pancreatitis (*n* = 2), hypertension (*n* = 3), osteoarthritis (*n* = 2), intervertebral disc disease (*n* = 1), epilepsy (*n* = 1), narrowly excised high‐grade soft tissue sarcoma (*n* = 1), incompletely excised grade 2 mast cell tumour (*n* = 1), vacuolar hepatopathy (*n* = 1), hyperadrenocorticism (*n* = 1), hypothyroidism (*n* = 1), bilateral keratoconjunctivitis sicca (*n* = 1), tracheal collapse (*n* = 1) and cranial cruciate ligament rupture (*n* = 1).
Concomitant medications included: famotidine (*n* = 5), omeprazole (*n* = 4), maropitant (*n* = 4), enalapril (*n* = 4), metronidazole (*n* = 3), ondansetron (*n* = 3), carprofen (*n* = 2), tramadol (*n* = 2), levetiracetam (*n* = 2), zonisamide (*n* = 2), gabapentin (*n* = 2), phenobarbital (*n* = 1), amlodipine (*n* = 2), mirtazapine (*n* = 1), loperamide (*n* = 1), psyllium fibre (*n* = 1), FortiFlora™ (*n* = 1), amoxicillin clavulanic acid (*n* = 1), enrofloxacin (*n* = 1), marbofloxacin (*n* = 1), s‐adenosylmethionine/silibyn (*n* = 1), ursodiol (*n* = 1), cetirizine (*n* = 1) and a cannabinoid oil (*n* = 1).

## Data Availability

All data relevant to the study are included in the article. The datasets used and analysed during the current study are available from the corresponding author upon reasonable request.
