# Supplementary material for: Toceranib phosphate in the management of canine insulinoma: A retrospective multicentre study of 30 cases (2009–2019)
Source: Vet Rec Open. 2022 Jan 20;9(1):e27. doi: 10.1002/vro2.27 (PMC8776903; doi:10.1002/vro2.27)
Supplement: Supplementary file 1 — Supporting Information S1 Further information results – Presentation, diagnosis and staging Haematological analyses were available for 25 dogs, and serum chemistry panels for 29, at the time of toceranib initiation. The haematological changes reported were all grade 1, including anaemia (n = 3), haemoconcentration (n = 1), neutropenia (n = 1), neutrophilia (n = 5), lymphopenia (n = 5), monocytosis (n = 1), thrombocytopenia (n = 1) and thrombocytosis (n = 2). The most commonly reported biochemical abnormality was hypoglycaemia (n = 25). Other biochemical changes reported included; variable elevations in alanine transferase (n = 4, grade 1 n = 2, grade 2 n = 2), variable elevations in alkaline phosphatase (n = 9, grade 1 n = 1, grade 2 n = 2, grade 3 n = 1, grade 4 n = 1 and ungraded n = 4), grade 1 hypercholesterolaemia (n = 2), grade 2 elevated blood urea nitrogen (n = 1), grade 1 elevated creatinine (n = 1), ungraded hypophosphataemia (n = 2), grade 1 hypokalaemia (n = 2) and ungraded elevations in both amylase (n = 4) and lipase (n = 2). Concurrent urinalyses were available for 14 dogs. Two dogs were hyposthenuric and one dog isosthenuric. No dogs were proteinuric. Six dogs had systolic blood pressure measurement performed. Three were hypertensive (systolic blood pressure >140 mmHg). Comorbidities included chronic pancreatitis (n = 2), hypertension (n = 3), osteoarthritis (n = 2), intervertebral disc disease (n = 1), epilepsy (n = 1), narrowly excised high‐grade soft tissue sarcoma (n = 1), incompletely excised grade 2 mast cell tumour (n = 1), vacuolar hepatopathy (n = 1), hyperadrenocorticism (n = 1), hypothyroidism (n = 1), bilateral keratoconjunctivitis sicca (n = 1), tracheal collapse (n = 1) and cranial cruciate ligament rupture (n = 1). Concomitant medications included: famotidine (n = 5), omeprazole (n = 4), maropitant (n = 4), enalapril (n = 4), metronidazole (n = 3), ondansetron (n = 3), carprofen (n = 2), tramadol (n = 2), levetiracetam (n = 2), zonisam [file VRO2-9-e27-s001.pdf]

Supporting Information

S1 Further information Results – Presentation, diagnosis and staging

Haematological analyses were available for 25 dogs, and serum chemistry panels for 29, at the time of toceranib initiation. The haematological changes reported were all grade 1, including anaemia (n=3), haemoconcentration (n=1), neutropenia (n=1), neutrophilia (n=5), lymphopenia (n=5), monocytosis (n=1), thrombocytopaenia (n=1) and thrombocytosis (n=2). The most commonly reported biochemical abnormality was hypoglycaemia (n=25). Other biochemical changes reported included; variable elevations in alanine transferase (n=4, grade 1 n=2, grade 2 n=2), variable elevations in alkaline phosphatase (n=9, grade 1 n=1, grade 2 n=2, grade 3 n=1, grade 4 n=1 and ungraded n=4), grade 1 hypercholesterolaemia (n=2), grade 2 elevated blood urea nitrogen (n=1), grade 1 elevated creatinine (n=1), ungraded hypophosphatemia (n=2), grade 1 hypokalaemia (n=2) and ungraded elevations in both amylase (n=4) and lipase (n=2). Concurrent urinalyses were available for 14 dogs. Two dogs were hyposthenuric and one dog isosthenuric. No dogs were proteinuric. Six dogs had systolic blood pressure measurement performed. Three were hypertensive (systolic blood pressure >140 mmHg).

Comorbidities included chronic pancreatitis (n=2), hypertension (n=3), osteoarthritis (n=2), intervertebral disc disease (n=1), epilepsy (n=1), narrowly-excised high grade soft tissue sarcoma (n=1), incompletely excised grade 2 mast cell tumour (n=1), vacuolar hepatopathy (n=1), hyperadrenocorticism (n=1), hypothyroidism (n=1), bilateral keratoconjunctivitis sicca (n = 1), tracheal collapse (n=1) and cranial cruciate ligament rupture (n=1).

Treatments – concurrent

Concomitant medications included; famotidine (n=5), omeprazole (n=4), maropitant (n=4), enalapril (n=4), metronidazole (n=3), ondansetron (n=3), carprofen (n=2), tramadol (n=2), levetiracetam (n=2), zonisamide (n=2), gabapentin (n=2), phenobarbital (n=1), amlodipine (n=2), mirtazapine (n=1), loperamide (n=1), psyllium fibre (n=1), FortiFlora™ (n=1), amoxicillin clavulanic acid (n=1), enrofloxacin (n=1), marbofloxacin (n=1), s-adenosylmethionine/silibyn (n=1), ursodiol (n=1), cetirizine (n=1) and a cannabinoid oil (n=1).
